# Maternal-Newborn ABO Blood Groups and Risk of Bacterial Infection in Newborns

**DOI:** 10.1001/jamanetworkopen.2024.42227

**Published:** 2024-10-30

**Authors:** Emily Ana Butler, Joel G. Ray, Eyal Cohen

**Affiliations:** 1 Child Health Evaluative Sciences, The Hospital for Sick Children, Toronto, Ontario, Canada; 2 ICES, Toronto, Ontario, Canada; 3 Department of Medicine, St Michael’s Hospital, Toronto, Ontario, Canada; 4 Edwin S.H. Leong Centre for Healthy Children, Department of Pediatrics, University of Toronto, Toronto, Ontario, Canada

## Abstract

**Importance:**

Newborn immunity largely relies on maternal-fetal transfer of antibodies in utero. Incongruency in ABO blood groups between a mother and newborn may be associated with protection against serious infections, but data specific to newborn bacterial infections are lacking.

**Objective:**

To ascertain the association between maternal-newborn ABO blood group incongruence and lower risk of bacterial infection in newborns.

**Design, Setting, and Participants:**

This cohort study used linked patient-level datasets for all singleton live births between January 1, 2014, and December 31, 2020, in hospitals and health centers in Ontario, Canada. The cohort comprised maternal-newborn pairs with known ABO blood groups. Data analysis was conducted between February and May 2024.

**Exposure:**

Maternal-newborn ABO blood group incongruence vs congruence.

**Main Outcomes and Measures:**

The primary outcome was a bacterial infection arising in newborns within 30 days of birth. Bacterial infection was cultured from either blood, cerebrospinal fluid, urine, or lung specimen. Secondary outcomes were a bacterial infection with 7 days and 90 days of birth. Modified Poisson regression generated adjusted relative risks (ARRs) and 95% CIs, adjusted for neonatal sex and preterm birth.

**Results:**

A total of 138 207 maternal-newborn pairs (maternal mean [SD] age, 31.8 [5.1] years among those with ABO blood group incongruency and 31.5 [5.1] years among those with ABO blood group congruency; newborn mean [SD] gestational age, 38.5 [2.3] weeks among those with incongruency and 38.4 [2.5] weeks among those with congruency; 19 475 males [51.3%] with incongruency and 52 041 males [51.9%] with congruency) were analyzed. Of these pairs, 37 953 (27.5%) had ABO blood group incongruency and 100 254 (72.5%) had ABO blood group congruency. Within 30 days of birth, 328 (8.6 per 1000) newborns in the incongruent group and 1029 (10.3 per 1000) newborns in the congruent group experienced a bacterial infection, corresponding to an ARR of 0.91 (95% CI, 0.81-1.03). The ARRs for bacterial infection within 7 days and 90 days of birth were 0.89 (95% CI, 0.73-1.09) and 0.86 (95% CI, 0.78-0.94), respectively.

**Conclusions and Relevance:**

This cohort study found no association between maternal-newborn ABO blood group incongruence and risk of bacterial infection in newborns within 30 and 7 days of birth. However, incongruence was associated with a decreased risk of bacterial infection within 90 days of birth.

## Key Points

**Question:**

If a mother and newborn have incongruent ABO blood groups, is the newborn at a lower risk of bacterial infection than if they had congruent ABO blood group?

**Findings:**

In this cohort study of 138 207 mother-newborn pairs, ABO blood group incongruence was not associated with a lower risk of bacterial infection within 30 days of birth.

**Meaning:**

Findings of this study suggest ABO blood group incongruence is not associated with a lower risk of bacterial infection within 30 days of birth.

## Teaser

This cohort study evaluates the risk of bacterial infection onset at 7, 30, and 90 days after birth of neonates with dissimilar ABO blood group from their mother.

## Introduction

The *ABO* gene indirectly encodes the ABO blood group antigen, projected via oligosaccharide chains on the surface of red blood cells, with 3 main allelic forms: A (A antigens present), B (B antigens present), and O (no antigens present).^1^ In human serum, immunoglobulin M (IgM) and immunoglobulin G (IgG) antibodies are produced that may oppose foreign A and B blood group antigens.^1,2^ Maternal IgG antibodies are transported across the placenta via an Fc receptor–mediated process, with the greatest transfer occurring during the third trimester of pregnancy.^3^ In utero, maternal-fetal antibody transfer is crucial for short-term passive fetal immunity, thereby protecting the infant against infection up to 6 months of age until their immune system is fully developed or they are vaccinated.^3,4,5^ The risk of bacterial infection is highest during the neonatal period,^6^ especially in the presence of untreated maternal Group B *Streptococcus* (GBS) infection, premature rupture of membranes, and preterm birth.^7^

It is of interest that the risk of bacterial infection may be related to ABO blood groups.^8^ Prior studies have shown a link between ABO blood type and newborn sepsis, with O blood group conferring protection and AB blood group revealing increased susceptibility to sepsis.^9,10^ However, no research has evaluated the association of neonatal sepsis from antibody transfer in utero with maternal and newborn ABO blood groups. If potentially protective IgG antibodies can be transferred from the pregnant individual to the fetus—such as the case when mother and offspring have dissimilar (ie, incongruent) ABO blood groups (eTable 1 in Supplement 1)—then a logical question is, does maternal-fetal ABO blood group incongruence confer a lower risk of bacterial infection on the newborn than maternal-fetal ABO blood group congruence? In a previous study, we found that ABO blood group incongruence between a mother and newborn is associated with protection against serious bacterial and viral infections,^11^ but details of the type and anatomical location of those bacterial infections are lacking.

The universal health care system in Ontario, Canada, enables the systematic linkage of all maternal and newborn health data, including ABO blood group status and bacterial culture results from blood, cerebrospinal fluid (CSF), urine, and lung samples. Accordingly, the current study aimed to ascertain the association between maternal-newborn ABO blood group incongruence and lower risk of bacterial infection in newborns.

## Methods

In this population-based retrospective cohort study, we considered all mothers with a singleton live birth in a hospital or community health center in Ontario, Canada, between January 1, 2014, and December 31, 2020. Excluded were non-Ontario residents, newborns with missing gestational age at birth, neonates with unknown birthweight, and mothers and/or neonates with unknown ABO blood group given that ABO blood group testing in Ontario is not routinely done for all newborns (eTable 2 and eFigure 1 in Supplement 1). Use of data in this project was authorized under section 45 of Ontario’s Personal Health Information Protection Act. This act allows for the use of routinely collected administrative data without consent and does not require the approval of a research ethics board. We followed the Reporting of Studies Conducted Using Observational Routinely Collected Data (RECORD) reporting guideline.^12^

All births were captured using existing Ontario patient-level databases that link unique maternal and newborn records. Each database is described in eTable 3 in Supplement 1.

### Exposures and Outcomes

The main exposure was maternal-newborn ABO blood group congruence, defined as a dichotomous exposure state of congruent or incongruent (eTable 1 in Supplement 1). In the incongruent state, the pregnant individual would be exposed to a fetal foreign A or B antigen and, therefore, would be more likely to produce an IgM or IgG antibody that could be transferred to the fetus and be present at birth and for many weeks thereafter.

The primary outcome was a bacterial infection arising within 30 days after birth. The secondary outcome was a bacterial infection arising within 7 days and 90 after birth. A bacterial infection was defined as a positive microbiological test result of blood, CSF, urine, or lung sample obtained in any hospital or community health center in Ontario.

### Statistical Analysis

Maternal and newborn characteristics were recorded as a number (%), mean (SD), or median (IQR), as appropriate. Standardized differences between congruent and incongruent ABO blood groups were assessed, with a value greater than 0.10 considered to be important.^13^ Separately, to assess for selection bias, the characteristics of newborns with a recorded ABO blood group were compared with characteristics of those without; then, the characteristics of newborns who had a microbiological sample drawn regardless of culture positivity were compared with characteristics of those without such samples. Because the databases did not include information on race and ethnicity, we used maternal world region of origin as a crude proxy for race and ethnicity, wherein we found no differences in ABO blood group congruence and incongruence.

In the main models, adjusted rate differences per 1000 newborns and 95% CIs were calculated for bacterial infections between incongruent and congruent ABO blood groups, with the latter group serving as the referent. Modified Poisson regression generated unadjusted and adjusted relative risks (ARR) and 95% CIs, with adjustment for neonatal sex and preterm birth before 37 weeks’ gestation.

In a second set of models, bacterial infections were separated into gram-positive or gram-negative bacterial organism among maternal-newborn pairs with incongruent vs congruent ABO blood groups. The analysis of gram-positive bacterial organisms was conducted with and without the inclusion of *Staphylococcus* species other than *Staphylococcus aureus*, as these organisms may be contaminants in cultures.

In additional analysis 1, the main model for 30-day bacterial infection was stratified by various factors associated with neonatal sepsis, including neonatal sex, mode of delivery, timing of birth, maternal GBS status, premature rupture of membranes, neonatal intensive care unit (NICU) admission, and maternal GBS antibiotics administration (eFigure 2 in Supplement 1).

Additional analysis 2 used multinomial logistic regression to analyze the association between maternal-newborn ABO blood group congruence and 4 possible outcomes: no culture organism, either with no microbiological sample collected or with a microbiological sample drawn and a negative culture result; gram-positive culture organism; gram-negative culture organism; or mixed-culture organism.

Additional analysis 3 considered the source of the microbiological testing: blood or CSF, urine, or lung. Similarly, additional analysis 4 grouped CSF, blood, and lung-based samples together, separate from urine samples. Given that death may precede the capturing of cultures, we expanded the outcome of additional analysis 5 to include a composite of bacterial infection or all-cause mortality within 30 days of birth.

All analyses were performed using SAS version 9.4 (SAS Institute Inc). Data analysis was conducted between February and May 2024.

## Results

A total of 138 207 maternal-newborn pairs (maternal mean [SD] age, 31.8 [5.1] years among those with ABO blood group incongruency and 31.5 [5.1] years among those with ABO blood group congruency; newborn mean [SD] gestational age, 38.5 [2.3] weeks among those with incongruency and 38.4 [2.5] weeks among those with congruency; 19 475 males [51.3%] and 18 478 females [48.7%] with incongruency; 52 041 males [51.9%] and 48 213 (48.1%] with congruency) were included in the main cohort. Of these pairs, 37 953 (27.5%) had incongruent ABO blood groups and 100 254 (72.5%) had congruent ABO blood groups (eFigure 1 in Supplement 1). Newborn characteristics at birth were largely similar between the congruent and incongruent groups except for neonatal jaundice or hyperbilirubinemia (9494 [9.5%] vs 4796 [12.6%]) and hemolytic disease of the newborn (312 [0.3%] vs 1985 [5.2%]) (Table 1).

**Table 1.** Characteristics of Mothers and Newborns in Congruent vs Incongruent ABO Blood Group

Characteristic	Congruent ABO blood group (n = 100 254)	Incongruent ABO blood group (n = 37 953)	Standardized difference
Maternal characteristics			
Age, mean (SD), y	31.5 (5.1)	31.8 (5.1)	0.05
Age by group, y			
≤19	1316 (1.3)	407 (1.1)	0.02
20-24	7735 (7.7)	2656 (7.0)	0.03
25-29	24 055 (24.0)	8740 (23.0)	0.02
30-34	38 857 (38.8)	14 928 (39.3)	0.01
35-39	22 920 (22.9)	8958 (23.6)	0.01
≥40	5371 (5.4)	2264 (6.0)	0.03
Gravidity, median (IQR)	1 (0-2)	1 (0-2)	0.02
Gravidity			
0	33 657 (33.6)	12 833 (33.8)	0.01
1	31 032 (31.0)	11 993 (31.6)	0.01
2	17 820 (17.8)	6562 (17.3)	0.01
≥3	17 714 (17.7)	6550 (17.3)	0.01
Unknown or missing data	31 (0.0)	15 (0.0)	0.01
Parity, median (IQR)	1 (0-1)	1 (0-1)	0.01
Parity			
0	46 483 (46.4)	17 589 (46.3)	0.00
1	35 145 (35.1)	13 581 (35.8)	0.02
2	12 407 (12.4)	4572 (12.0)	0.01
≥3	6219 (6.2)	2211 (5.8)	0.02
World region of origin			
Canada^a^	69 221 (69.0)	24 787 (65.3)	0.08
Caribbean or Africa	3520 (3.5)	1306 (3.4)	0.00
East Asia or Pacific	8634 (8.6)	3943 (10.4)	0.06
Hispanic America	2168 (2.2)	794 (2.1)	0.01
Middle East or North Africa	4242 (4.2)	1933 (5.1)	0.04
South Asia	7653 (7.6)	3141 (8.3)	0.02
Western Nations or Europe	4816 (4.8)	2049 (5.4)	0.03
Residential income quintile			
Quintile 1 (lowest) or unknown	20 341 (20.3)	7625 (20.1)	0.01
Quintile 2	19 134 (19.1)	7226 (19.0)	0.00
Quintile 3	21 099 (21.0)	7870 (20.7)	0.01
Quintile 4	22 584 (22.5)	8642 (22.8)	0.01
Quintile 5 (highest)	17 096 (17.1)	6590 (17.4)	0.01
Rural residence	7246 (7.2)	2245 (5.9)	0.05
ABO blood group			
A	26 040 (26.0)	3246 (8.6)	0.47
B	11 484 (11.5)	3047 (0.1)	0.12
AB	4423 (4.4)	0	0.30
O	58 307 (58.2)	31 660 (83.4)	0.58
Rh negative	25 747 (25.7)	6913 (18.2)	0.18
Maternal conditions within 365 d prior to conception			
Type 1 or type 2 diabetes	123 (0.1)	49 (0.1)	0.00
Chronic hypertension	74 (0.1)	20 (0.1)	0.01
Sickle cell disease	172 (0.2)	71 (0.2)	0.00
Prepregnancy BMI group^b^			
Underweight (<18.5)	4115 (4.1)	1605 (4.2)	0.01
Healthy weight (18.5-24.9)	41 200 (41.1)	15 980 (42.1)	0.02
Overweight (25.0-29.9)	19 812 (19.8)	7280 (19.2)	0.02
Obesity (≥30)	16 140 (16.1)	5645 (14.9)	0.03
Unknown or missing data	18 987 (18.9)	7443 (19.6)	0.02
Tobacco or substance use	432 (0.4)	138 (0.4)	0.01
Any autoimmune disorder	708 (0.7)	232 (0.6)	0.01
Maternal conditions during the index pregnancy			
Gestational diabetes among mothers without type 1 or type 2 diabetes	8781 (8.8)	3496 (9.2)	0.02
Preeclampsia	1150 (1.1)	369 (1.0)	0.02
Rh factor incompatibility	83 (0.1)	7 (0.0)	0.03
Placenta previa	946 (0.9)	305 (0.8)	0.00
Premature rupture of membranes	15 215 (15.2)	5560 (14.6)	0.02
GBS positive result^c^	17 977 (17.9)	6669 (17.6)	0.01
GBS antibiotics	18 281 (18.2)	6677 (17.6)	0.02
Postpartum hemorrhage	5667 (5.7)	2277 (6.0)	0.02
Mode of delivery			
Vaginal	70 699 (70.5)	26 736 (70.4)	0.00
Cesarean	29 555 (29.5)	11 217 (29.6)	0.00
Newborn characteristics at index birth			
Sex			
Male	52 041 (51.9)	19 475 (51.3)	0.01
Female	48 213 (48.1)	18 478 (48.7)	0.01
Gestational age, mean (SD), wk	38.4 (2.5)	38.5 (2.3)	0.05
Gestational age group, wk			
≤27	1150 (1.1)	335 (0.9)	0.03
28-31	1910 (1.9)	522 (1.4)	0.04
32-33	1470 (1.5)	451 (1.2)	0.02
34-36	7065 (7.0)	2443 (6.4)	0.02
≥37	88 659 (88.4)	34 202 (90.1)	0.05
Birthweight, mean (SD), g	3258 (656)	3282 (619)	0.04
Apgar score at 5 min, median (IQR)^d^	9 (9-9)	9 (9-9)	
Apgar score at 5 min, by group^d^			
0-3	510 (0.5)	173 (0.5)	0.01
4-7	2290 (2.3)	723 (1.9)	0.03
≥7	94 642 (94.4)	35 926 (94.7)	0.01
Unknown or missing data	2812 (2.8)	1131 (3.0)	0.05
Hereditary immunodeficiency	19 (0.0)	<6^e^	0.01
Neonatal jaundice or hyperbilirubinemia	9494 (9.5)	4796 (12.6)	0.10
Hemolytic disease of the newborn	312 (0.3)	1985 (5.2)	0.30
Admitted to NICU	17 521 (17.5)	5869 (15.5)	0.05
ABO blood group			
A	19 769 (19.7)	22 051 (58.1)	0.86
B	9100 (9.1)	12 456 (32.8)	0.61
AB	815 (0.8)	3446 (9.1)	0.39
O	70 570 (70.4)	0	2.18
Rh positive	85 217 (85.0)	33 186 (87.4)	0.07

Abbreviations: BMI, body mass index (calculated as weight in kilograms divided by height in meters squared); GBS, group B *Streptococcus*; NICU, neonatal intensive care unit.

^a^ Includes long-term residents of Canada for more than 10 years.

^b^ Comprises 111 777 births (80.9%) with known maternal prepregnancy BMI data.

^c^ Comprises 111 275 births (80.9%) with known GBS status.

^d^ Comprises 134 264 births (97.2%) with known Apgar score at 5 minutes.

^e^ Fewer than 6 events are suppressed.

Comparing newborns with vs without available ABO blood group information showed that rates of neonatal jaundice or hyperbilirubinemia were higher among those with data (14 290 [10.3%] vs 31 095 [5.3%]), as were rates of hemolytic disease of the newborn (2297 [1.7%] vs 3400 [0.6%]) and NICU admission (23 390 [16.9%] vs 65 981 [11.2%]) (eTable 4 in Supplement 1). Otherwise, there were no notable differences among newborns or mothers with or without ABO blood group information. Separately, among newborns with vs without a microbiological sample drawn, NICU admission was much higher among newborns who had their sample drawn (8834 [75.4%] vs 14 556 [11.5%]) (eTable 5 in Supplement 1).

The primary outcome of bacterial infection within 30 days of birth occurred in 328 (8.6 per 1000) newborns in the incongruent ABO blood group compared with 1029 (10.3 per 1000) newborns in the congruent group. The corresponding adjusted rate difference was −0.6 (95% CI, −1.3 to 0.2) per 1000 newborns and an ARR of 0.91 (95% CI, 0.81-1.03) (Table 2).

**Table 2.** Risk of Bacterial Infection in Newborns in Congruent vs Incongruent ABO Blood Groups

Study outcome and exposure	No. of newborns with outcome (rate per 1000 newborns)	Adjusted rate difference, rate per 1000 (95% CI)^a^	RR (95% CI)	
			Unadjusted	Adjusted RR^a^
Primary outcome: bacterial infection within 30 d of birth				
Congruent ABO blood groups (n = 100 254)	1029 (10.3)	0 [Reference]	1 [Reference]	1 [Reference]
Incongruent ABO blood groups (n = 37 953)	328 (8.6)	−0.6 (−1.3 to 0.2)	0.84 (0.74 to 0.95)	0.91 (0.81 to 1.03)
Secondary outcome: bacterial infection within 7 d of birth				
Congruent ABO blood groups (n = 100 254)	398 (4.0)	0 [Reference]	1 [Reference]	1 [Reference]
Incongruent ABO blood groups (n = 37 953)	125 (3.3)	−0.3 (−0.8 to 0.2)	0.83 (0.68 to 1.01)	0.89 (0.73 to 1.09)
Secondary outcome: bacterial infection within 90 d of birth				
Congruent ABO blood groups (n = 100 254)	1834 (18.3)	0 [Reference]	1 [Reference]	1 [Reference]
Incongruent ABO blood groups (n = 37 953)	561 (14.8)	−1.9 (−3.0 to −0.8)	0.81 (0.74 to 0.89)	0.86 (0.78 to 0.94)

Abbreviation: RR, relative risk.

^a^ Adjusted for neonatal sex and preterm birth before 37 weeks’ gestation.

There was a lower risk of infection among full-term newborns with incongruent ABO blood groups compared with the congruent group (Figure). This lower risk was particularly notable among newborns whose mother had negative GBS culture (ARR, 0.80; 95% CI, 0.65-0.99) and in the absence of premature rupture of membranes (ARR, 0.85; 95% CI, 0.74-0.99) (Figure).

**Figure.**
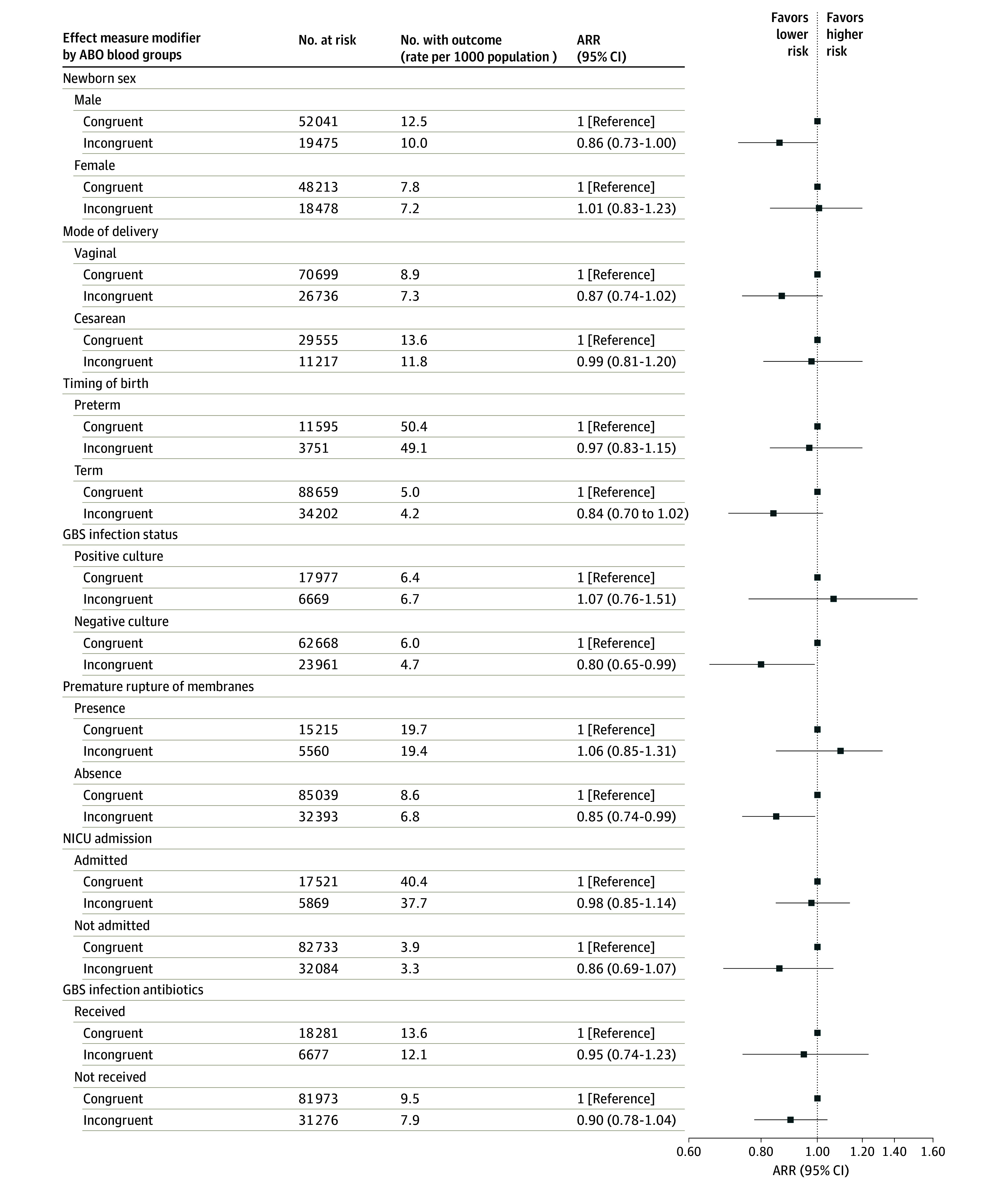
Risk of Bacterial Infection in Newborns Within 30 Days of Birth Stratified by Effect Measure Modifiers in Maternal-Newborn Incongruent vs Congruent ABO Blood Group Error bars represent 95% CIs. ARR indicates adjusted relative risk; GBS, group B *Streptococcus*; NICU, neonatal intensive care unit.

The secondary outcome of a bacterial infection within 7 days of birth occurred in 125 (3.3 per 1000) newborns in the incongruent ABO blood group vs 398 (4.0 per 1000) newborns in the congruent group (ARR, 0.89; 95% CI, 0.73-1.09) (Table 2). A bacterial infection within 90 days of birth occurred in 561 (14.8 per 1000) infants in the incongruent group vs 1834 (18.3 per 1000) infants in the congruent group (ARR, 0.86; 95% CI, 0.78-0.94) (Table 2).

The top 10 most common gram-positive bacterial species among newborns included *Staphylococcus* species other than *S aureus* (268 [39.9%]), *Enterococcus* species (95 [13.6%]), and *S aureus* (89 [13.4%]) (eTable 6 in Supplement 1). The top 10 gram-negative bacterial species included *Escherichia coli* (296 [58.7%]), *Klebsiella* species (74 [14.7%]), and *Enterobacter* species (53 [10.5%]) (eTable 6 in Supplement 1). ABO blood group incongruence was associated with an ARR of 0.85 (95% CI, 0.71-1.02) for gram-positive bacterial infection within 30 days of birth and 0.96 (95% CI, 0.79-1.17) for gram-negative bacterial infection in the newborn (Table 3). After excluding *Staphylococcus* species other than *S aureus*, the ARR was 0.86 (95% CI, 0.68-1.09).

**Table 3.** Risk of Bacterial Infection in Newborns Within 30 Days of the Index Birth by Bacterial Gram Stain in Congruent vs Incongruent ABO Blood Groups

Infection type and risk factor	No. of newborns with outcome (rate per 1000 newborns)	RR (95% CI)	
		Unadjusted	Adjusted RR^a^
Gram-positive bacterial infection			
Congruent ABO blood groups (n = 100 254)	512 (5.1)	1 [Reference]	1 [Reference]
Incongruent ABO blood groups (n = 37 953)	151 (4.0)	0.78 (0.65 to 0.93)	0.85 (0.71 to 1.02)
Gram-negative bacterial infection			
Congruent ABO blood groups (n = 100 254)	377 (3.8)	1 [Reference]	1 [Reference]
Incongruent ABO blood groups (n = 37 953)	127 (3.4)	0.89 (0.73 to 1.08)	0.96 (0.79 to 1.17)

Abbreviation: RR, relative risk.

^a^ Adjusted for neonatal sex and preterm birth before 37 weeks’ gestation.

Compared with newborns who did not have a microbiological sample collected, the adjusted odds ratio was 0.85 (95% CI, 0.71-1.02) for a gram-positive culture organism (eTable 7 in Supplement 1). For gram-negative culture organisms and mixed-culture organisms, no association was observed.

The most common specimen sites for a bacterial infection in newborns were the urine (582 [42.9%]), blood (480 [35.4%]), and endotracheal tube (221 [16.3%]) (eTable 8 in Supplement 1). The ARR was 0.88 (95% CI, 0.72-1.07) for a bacterial infection in the blood or CSF in the presence of ABO blood group incongruence (Table 4). The associated risk was less pronounced in urine (ARR, 0.95; 95% CI, 0.79-1.14) and lung (ARR, 0.91; 95% CI, 0.67-1.23) cultures (Table 4). After separating culture-positive CSF, blood, and lung microbiological samples from those with a culture-positive urine specimen, there was a lower risk of infection among newborns in the incongruent ABO blood group (eTable 9 in Supplement 1). Expanding the study outcome to bacterial infection or death within 30 days of birth showed an ARR of 0.94 (95% CI, 0.83-1.05) in newborns with ABO blood group incongruence (eTable 10 in Supplement 1).

**Table 4.** Risk of Bacterial Infection in Newborns Within 30 Days of the Index Birth by Sample Source in Congruent vs Incongruent ABO Blood Groups

Microbiological sample source and risk factor	No. of newborns with outcome (rate per 1000 newborns)	RR (95% CI)	
		Unadjusted	Adjusted RR^a^
Blood or cerebrospinal fluid			
Congruent ABO blood groups (n = 100 254)	421 (4.2)	1 [Reference]	1 [Reference]
Incongruent ABO blood groups (n = 37 953)	128 (3.4)	0.80 (0.66 to 0.98)	0.88 (0.72 to 1.07)
Urine			
Congruent ABO blood groups (n = 100 254)	434 (4.3)	1 [Reference]	1 [Reference]
Incongruent ABO blood groups (n = 37 953)	148 (3.9)	0.90 (0.75 to 1.09)	0.95 (0.79 to 1.14)
Lung			
Congruent ABO blood groups (n = 100 254)	174 (1.7)	1 [Reference]	1 [Reference]
Incongruent ABO blood groups (n = 37 953)	52 (1.4)	0.79 (0.58 to 1.08)	0.91 (0.67 to 1.23)

Abbreviation: RR, relative risk.

^a^ Adjusted for infant sex and gestational age at birth (in weeks).

## Discussion

In this population-based cohort study, maternal-newborn ABO blood group incongruence was not associated with a lower risk of bacterial infection within 30 days of birth (primary outcome) or within 7 days of birth (secondary outcome). Incongruent ABO blood group was associated with a lower risk of bacterial infection within 90 days of birth (secondary outcome) among infants with lower risk states, including absence of premature rupture of membranes, and infants whose mother had a negative GBS culture.

### Study Mechanisms

The most pronounced association between ABO blood group incongruence and infant infection was at 90 days from birth but not at 30 days, the time when passively transferred antibodies from mother to newborn were at the highest level. Perhaps the differing ABO antibodies that transferred in utero from a pregnant individual with incongruent blood group to the fetus played a less important role in overall newborn immunity than other passively transferred antibodies in the immediate period after birth, as the newborn’s immune system is gradually built over many weeks or months after birth.^5,14^ Although 42.9% of infections were urinary, no appreciable association was seen with ABO blood group incongruence. One explanation may be that urinary infections are typically less dependent on host humoral immunity.^15,16^

The apparent protection against bacterial infection associated with ABO blood group incongruence was not observed among preterm newborns, newborns of mothers with positive GBS culture, or newborns with premature rupture of membranes. One explanation is that antibiotics are often empirically administered at birth to preterm newborns^17^ and to mothers with positive GBS culture or premature rupture of membranes,^18,19^ thereby masking the detection of any protection associated with ABO blood group incongruence in the newborn. Perhaps only after some time would blunting from perinatally administered antibiotics wane and the protection associated with incongruent ABO blood group become apparent by 90 days after birth.

In contrast to our prior study,^11^ the current study’s findings specifically assessed cultured bacterial infections. By requiring that the current outcome be based on bacterial culture positivity, we may have lost sensitivity for detecting true neonatal sepsis in the absence of bacterial specimen collection or culture positivity, thereby blunting any true association.

### Future Research

Because differing levels of antibodies in the pregnant individual and the fetus may affect the efficiency of antibody transfer across the placenta, a future study could measure maternal ABO IgG antibody titers during pregnancy and in the infant at birth and over the ensuing months. In current clinical practice, whole blood samples are typically required only to conduct a Coomb test among newborns with jaundice^20^ or if the newborn is ill. These already available blood samples could also be used to test for antibody titers, with another sample collected beyond the newborn period, thus providing critical insight into the temporal relationship between maternal and infant antibody levels and immune protection. This approach may clarify any relationship between anti–A and anti–B antibodies at birth, resulting from placental antibody transfer, and subsequent risk of serious infection in the newborn and infant.

Future clinical research should explore ABO blood group incompatibility as a variable in the clinical estimation of bacterial infection in the newborn. Moreover, future work can assess whether race and ethnicity modify any association between ABO blood group incongruence and neonatal bacterial infection risk.

### Strengths and Limitations

This study analyzed a large population-based sample of hospital live births in Ontario. The universal health care in this Canadian province permitted the collection of health information on maternal-newborn pairs, such as ABO blood group status and microbiological samples.

A major study limitation is that approximately 90% of newborns lacked ABO blood group data. In Ontario, a newborn’s blood type is generally tested if the mother is Rh negative or if the newborn is severely ill.^21,22,23^ Even so, newborns with and without known ABO blood group status differed minimally, except for hyperbilirubinemia, hemolytic diseases, and NICU admission. Separately, we lacked information on the indication for microbiological sampling or the ability to differentiate colonization from infection. However, newborns who had microbiological testing differed minimally from those who did not have testing, with few exceptions such as earlier birth and NICU admission—factors that can predispose to infection and therefore potentially introduce participant selection bias. Additionally, the databases we used did not include information on antibiotic type and duration, administration of immune-modulating therapies (eg, immunoglobulin), or breastfeeding practices. Breastfeeding permits the early transfer of IgM antibodies, playing a role in infant passive immunity and capacity to fight infection.^24,25^ The databases also did not include information on race and ethnicity.

## Conclusions

In this cohort study, maternal-newborn ABO blood group incongruence was not associated with a lower risk of infection within 30 or 7 days of birth. However, there was an association between incongruence and a decreased risk of bacterial infection in infants within 90 days after birth.

## Notes

Supplement 1. **eTable 1.** Mother and Newborn ABO Blood Groups, and Classification of Maternal-Infant Congruence or Incongruence **eTable 2.** Cohort Entry and Exclusion Criteria, and Methods and Coding to Identify Study Outcomes **eTable 3.** Description of the ICES Databases Used in the Current Study **eTable 4.** Characteristics of the Mothers and Their Singleton Neonates At Birth, Contrasting Neonates With Known vs. Unknown ABO Blood Group Status **eTable 5.** Characteristics of the Mothers and Their Singleton Neonates At Birth, Contrasting Neonates With a Microbiology Sample Taken vs. Those Who Did Not **eTable 6.** The Top-10 Gram-Positive and Gram-Negative Bacterial Species Among Neonates With a Positive Bacterial Culture From a Blood, Cerebral Spinal Fluid, Lung, or Urine Sample Obtained Within 30 Days of Birth **eTable 7.** Additional Analysis 2: Odds of an Invasive Gram-Positive or Gram-Negative Newborn Bacterial Infection Within 30 Days After Birth in Relation to Maternal-Newborn Incongruent vs. Congruent ABO Blood Groups **eTable 8.** Number of Invasive Newborn Bacterial Infections Diagnosed Within 30 Days After the Birth by Specimen Location **eTable 9.** Additional Analysis 4: Risk of Invasive Newborn Bacterial Infection Within 30 Days of the Index Birth in Relation to Maternal-Newborn Incongruent vs Congruent ABO Blood Groups, by Culture Source **eTable 10.** Additional Analysis 5: Risk of the Composite Outcome of Newborn Bacterial Infection or Death Within 30 Days of Birth, in Relation to Maternal-Newborn Incongruent vs Congruent ABO Blood Groups **eFigure 1.** Flow Diagram Showing Cohort Creation **eFigure 2.** Conceptual Model of Effect Measure Modifiers and Other Variables

Supplement 2.
Data Sharing Statement
