# Supplementary material for: Maternal-Newborn ABO Blood Groups and Risk of Bacterial Infection in Newborns
Source: JAMA Netw Open. 2024 Oct 30;7(10):e2442227. doi: 10.1001/jamanetworkopen.2024.42227 (PMC11525604; doi:10.1001/jamanetworkopen.2024.42227)
Supplement: Supplement 1. — eTable 1. Mother and Newborn ABO Blood Groups, and Classification of Maternal-Infant Congruence or Incongruence eTable 2. Cohort Entry and Exclusion Criteria, and Methods and Coding to Identify Study Outcomes eTable 3. Description of the ICES Databases Used in the Current Study eTable 4. Characteristics of the Mothers and Their Singleton Neonates At Birth, Contrasting Neonates With Known vs. Unknown ABO Blood Group Status eTable 5. Characteristics of the Mothers and Their Singleton Neonates At Birth, Contrasting Neonates With a Microbiology Sample Taken vs. Those Who Did Not eTable 6. The Top-10 Gram-Positive and Gram-Negative Bacterial Species Among Neonates With a Positive Bacterial Culture From a Blood, Cerebral Spinal Fluid, Lung, or Urine Sample Obtained Within 30 Days of Birth eTable 7. Additional Analysis 2: Odds of an Invasive Gram-Positive or Gram-Negative Newborn Bacterial Infection Within 30 Days After Birth in Relation to Maternal-Newborn Incongruent vs. Congruent ABO Blood Groups eTable 8. Number of Invasive Newborn Bacterial Infections Diagnosed Within 30 Days After the Birth by Specimen Location eTable 9. Additional Analysis 4: Risk of Invasive Newborn Bacterial Infection Within 30 Days of the Index Birth in Relation to Maternal-Newborn Incongruent vs Congruent ABO Blood Groups, by Culture Source eTable 10. Additional Analysis 5: Risk of the Composite Outcome of Newborn Bacterial Infection or Death Within 30 Days of Birth, in Relation to Maternal-Newborn Incongruent vs Congruent ABO Blood Groups eFigure 1. Flow Diagram Showing Cohort Creation eFigure 2. Conceptual Model of Effect Measure Modifiers and Other Variables [file jamanetwopen-e2442227-s001.pdf]

Supplemental Online Content

Butler EA, Ray JG, Cohen E. Maternal-newborn ABO blood groups and risk of bacterial infection in newborns. *JAMA Netw Open*. 2024;7(10):e2442227. doi:10.1001/jamanetworkopen.2024.42227

eTable 1. Mother and Newborn ABO Blood Groups, and Classification of Maternal-Infant Congruence or Incongruence

eTable 2. Cohort Entry and Exclusion Criteria, and Methods and Coding to Identify Study Outcomes

eTable 3. Description of the ICES Databases Used in the Current Study

eTable 4. Characteristics of the Mothers and Their Singleton Neonates At Birth, Contrasting Neonates With Known vs. Unknown ABO Blood Group Status

eTable 5. Characteristics of the Mothers and Their Singleton Neonates At Birth, Contrasting Neonates With a Microbiology Sample Taken vs. Those Who Did Not

eTable 6. The Top-10 Gram-Positive and Gram-Negative Bacterial Species Among Neonates With a Positive Bacterial Culture From a Blood, Cerebral Spinal Fluid, Lung, or Urine Sample Obtained Within 30 Days of Birth

eTable 7. Additional Analysis 2: Odds of an Invasive Gram-Positive or Gram-Negative Newborn Bacterial Infection Within 30 Days After Birth in Relation to Maternal-Newborn Incongruent vs. Congruent ABO Blood Groups

eTable 8. Number of Invasive Newborn Bacterial Infections Diagnosed Within 30 Days After the Birth by Specimen Location

eTable 9. Additional Analysis 4: Risk of Invasive Newborn Bacterial Infection Within 30 Days of the Index Birth in Relation to Maternal-Newborn Incongruent vs. Congruent ABO Blood Groups, by Culture Source

eTable 10. Additional Analysis 5: Risk of the Composite Outcome of Newborn Bacterial Infection or Death Within 30 Days of Birth, in Relation to Maternal-Newborn Incongruent vs. Congruent ABO Blood Groups

© 2024 Butler EA et al. *JAMA Network Open*

eFigure 1. Flow Diagram Showing Cohort Creation

eFigure 2. Conceptual Model of Effect Measure Modifiers and Other Variables

This supplemental material has been provided by the authors to give readers additional information about their work.

eTable 1. Mother and Newborn ABO Blood Groups, and Classification of Maternal-Infant Congruence or Incongruence

Incongruence is defined here as a state in which the newborn has a foreign A or B antigen that is not present in the mother, and hence the exposed mother may produce an IgM or IgG antibody response.

		Newborn			
	**ABO blood group**	**A**	**B**	**AB**	**O**
**Mother**	**A**	Congruent	Incongruent	Incongruent	Congruent
	**B**	Incongruent	Congruent	Incongruent	Congruent
	**AB**	Congruent	Congruent	Congruent	Not possible
	**O**	Incongruent	Incongruent	Not possible	Congruent

© 2024 Butler EA et al. *JAMA Network Open*

eTable 2. Cohort Entry and Exclusion Criteria, and Methods and Coding to Identify Study Outcomes

Assessment	Timing	Disease, procedure, or measure	ICD-10-CA diagnostic codes in DAD, MOMBABY and NACRS	Diagnostic Codes in OHIP (or other sources)
Cohort entry criteria	From one year prior to the index birth up to and including 365 days after the index birth from January 1, 2014 to December 31, 2020	All obstetrically delivered mothers and their linked newborns (livebirths and stillbirths) in the province of Ontario	Main patient service code indicating "obstetrical delivery" (the MOMBABY dataset includes linked DAD inpatient admission records of delivering mothers and their newborns; https://datadictionary.ices.on.ca/Applications/DataDictionary/Library.aspx?Library=MOMBABY)	
Exclusion criteria	From one year prior to the index birth up to and including 365 days after the index birth from January 1, 2014 to December 31, 2020	Woman or infant had an invalid healthcare number or hospital number	Invalid healthcare number on the DAD delivery record, or invalid maternal-newborn linkage in MOMBABY	Sex not 'Female', birth date missing or invalid, or unregistered healthcare number (RPDB)
	Same as above	Woman was a non-Ontario resident at index birth hospitalization		Postal code (RPDB)
	Same as above	Infant was not OHIP eligible at time of index birth		Eligibility start and end dates (RPDB)
	Same as above	Woman was not OHIP eligible one year prior to index birth, and/or during the index birth hospitalization		Eligibility start and end dates (RPDB)
	Same as above	Woman's age was missing or < 10 or > 55 years		Age (RPDB)
	At the index birth	Length of gestation missing, < 20 weeks or > 42 weeks	Clinical gestation weeks at delivery (from the DAD newborn record, if present, otherwise from the DAD delivery record), M_GESTWKS_DEL (MOMBABY)	

© 2024 Butler EA et al. *JAMA Network Open*

Assessment	Timing	Disease, procedure, or measure	ICD-10-CA diagnostic codes in DAD, MOMBABY and NACRS	Diagnostic Codes in OHIP (or other sources)
	Same as above	Multifetal delivery	ICD-10-CA (DAD delivery record): Z37.2-Z37.7, Z37.90, O30, O31<br>ICD-10-CA (DAD newborn record): Z38.3-Z38.6, Q89.4	
	Same as above	Liveborn infant with unknown birthweight	Newborn weight (DAD newborn record) B_WEIGHT from MOMBABY	
	Same as above	Liveborn infant discharged alive and had an invalid healthcare number	Discharged alive and invalid healthcare number (DAD newborn record)	Sex missing, birth date missing, or unregistered healthcare number (RPDB)
Main exposure	At the index birth	ABO blood group congruence between mother and newborn		LOINC codes 882-1, 883-9, 10331-7 in the Ontario Laboratory Information System (OLIS) - includes most outpatient laboratory information in Ontario
Main outcomes	From the index birth to 30 days after the index birth	Invasive bacterial infection diagnosed from a blood, CSF, lung, or urine microbiology culture		OLIS microbiology records

© 2024 Butler EA et al. *JAMA Network Open*

Assessment	Timing	Disease, procedure, or measure	ICD-10-CA diagnostic codes in DAD, MOMBABY and NACRS	Diagnostic Codes in OHIP (or other sources)
Secondary outcomes	From the index birth to 7 days after the index birth	Invasive bacterial infection diagnosed from a blood, CSF, lung, or urine microbiology culture		OLIS microbiology records
	From the index birth to 90 days after the index birth	Invasive bacterial infection diagnosed from a blood, CSF, lung, or urine microbiology culture		OLIS microbiology records
	From the index birth to 30 days after the index birth	Gram-positive or Gram-negative infection, diagnosed from a blood, CSF, lung or urine microbiology culture		OLIS microbiology records
Covariates (maternal)	From one year prior to the index birth up to and including 365 days after the index birth from January 1 2014 to December 31 2020	Age		Age (RPDB)
	Same as above	Parity	Previous term deliveries + Previous pre-term deliveries + Previous spontaneous abortions + Previous therapeutic abortions (DAD delivery record)	

© 2024 Butler EA et al. *JAMA Network Open*

Assessment	Timing	Disease, procedure, or measure	ICD-10-CA diagnostic codes in DAD, MOMBABY and NACRS	Diagnostic Codes in OHIP (or other sources)
	Same as above	World region of origin		FCOB – country of birth (CIC)
	Same as above	Residential income quintile		Statistics Canada Census
	Same as above	Urban residence		Statistics Canada Census
	Same as above	Rh factor		LOINC codes 882-1, 883-9, 10331-7 in the Ontario Laboratory Information System (OLIS) - includes most outpatient laboratory information in Ontario
	Same as above	ABO blood group		LOINC codes 882-1, 883-9, 10331-7 in the Ontario Laboratory Information System (OLIS) - includes most outpatient laboratory information in Ontario
	From within one year prior to conception	Type 1 or type 2 diabetes mellitus	ICD-10-CA (DAD): E107.8, E10.9, E117.8, E119	
	Same as above	Chronic hypertension	ICD-10-CA (DAD, SDS):<br>I10-I13, I15	
	Same as above	Sickle cell disease	ICD-10-CA (DAD): D57	
	Same as above	Body mass index		M0019 – Maternal BMI (BORN)
	Same as above	Tobacco or substance use	ICD-10-CA (DAD): Z72.0, Z72.1, Z72.2	
	Same as above	Any autoimmune disorder		M0013 – maternal health condition (BORN)

© 2024 Butler EA et al. *JAMA Network Open*

Assessment	Timing	Disease, procedure, or measure	ICD-10-CA diagnostic codes in DAD, MOMBABY and NACRS	Diagnostic Codes in OHIP (or other sources)
	In the index pregnancy	Gestational diabetes mellitus amount women without type 1 or type 2 diabetes mellitus	ICD-10-CA (DAD): O2480.1-4, O2480.9	
	Same as above	Preeclampsia	ICD-10-CA (DAD): O1400.1-4, O1400.9, O1410.1-4, O1410.9	
	Same as above	Placenta previa		M0531 – complication in pregnancy (BORN)
	Same as above	Postpartum hemmorhage	ICD-10-CA (DAD): O7200.2, O7200.4, O7200.9, O7210.2, O7210.4, O7210.9, O7220.2, O7220.4, O7220.9	
	Same as above	Maternal Group B streptococcus status		M0527 - Group B Strep screening results (BORN)
	Same as above	Maternal premature rupture of membranes	ICD-10-CA (DAD): O42	
	Same as above	Maternal Group B streptococcus antibiotics		M0528 - Group B Strep Antibiotics (BORN)
Covariates (newborn)	At index birth	Infant's date of birth	B_BDATE – baby's date of birth (MOMBABY)	
	Same as above	Gestational age at birth	B_GESTWKS_DEL – baby's gestational weeks at birth (MOMBABY)	
	Same as above	Birthweight	B_WEIGHT – baby's weight in grams at birth (MOMBABY)	
	Same as above	Sex	B_SEX – baby's sex on birth record (MOMBABY)	
	Same as above	Apgar score at 5 minutes		N0009 – APGAR score at 5 mins

© 2024 Butler EA et al. *JAMA Network Open*

Assessment	Timing	Disease, procedure, or measure	ICD-10-CA diagnostic codes in DAD, MOMBABY and NACRS	Diagnostic Codes in OHIP (or other sources)
	Same as above	Any neonatal adverse outcomes indicator during the index birth hospitalization		D0152 – neonatal health conditions (BORN)
	Same as above	Admitted to NICU		D0137 – NICU Admit date (BORN)
	Same as above	Presence of autoimmune disorders		D0152 – neonatal health conditions (BORN)
	Same as above	Rh factor		LOINC codes 882-1, 883-9, 10331-7 in the Ontario Laboratory Information System (OLIS) - includes most outpatient laboratory information in Ontario
	Same as above	ABO blood group		LOINC codes 882-1, 883-9, 10331-7 in the Ontario Laboratory Information System (OLIS) - includes most outpatient laboratory information in Ontario
	Same as above	Hemolytic disease of the newborn	ICD-10-CA (DAD): P55.0, P55.1, P55.8, P55.9	
	Same as above	Neonatal jaundice or hyperbilirubinemia	ICD-10-CA (DAD): P58.0-5, P58.8-9, P59.0-3, P59.8-9	
	Same as above	Hereditary immunodeficiency	ICD-10-CA (DAD): D80-D89, D72.0, M30.3, M35.9	

© 2024 Butler EA et al. *JAMA Network Open*

Assessment	Timing	Disease, procedure, or measure	ICD-10-CA diagnostic codes in DAD, MOMBABY and NACRS	Diagnostic Codes in OHIP (or other sources)
	Same as above	Mode of delivery	ICD-10-CA (DAD delivery record): O80, O81, O82, O83	

© 2024 Butler EA et al. *JAMA Network Open*

eTable 3. Description of the ICES Databases Used in the Current Study

Dataset name	Description
Canadian Institute for Health Information Discharge Abstract Database (CIHI-DAD)	Contains administrative, clinical (diagnoses and procedures/interventions), demographic, and administrative information for all admissions to acute care hospitals in Ontario. Diagnostic codes are captured using the International Statistical Classification of Diseases and Related Health Problems, 10th Revision, Canada (ICD-10-CA) coding system and interventions (up to 20 on a given DAD record) are captured using the Canadian Classification of Health Interventions (CCI) coding system.
National Ambulatory Care Reporting System (NACRS)	Contains administrative, clinical (diagnoses and procedures), demographic, and administrative information for all Ontarian patient visits made to hospital- and community-based ambulatory care centres (emergency departments, day surgery units, hemodialysis units, and cancer care clinics).
Ontario Health Insurance Plan (OHIP) Claims History Database	Contains information on inpatient and outpatient services provided to Ontario residents eligible for the province's publicly funded health insurance system by fee-for-service health care practitioners (primarily physicians) and "shadow billings" for those paid through non-fee-for-service payment plans.
Registered Persons Database (RPDB)	Provides basic demographic information for those issued an Ontario health insurance number, and indicates the time periods for which an individual was eligible to receive publicly funded health insurance benefits and the best-known postal code for each registrant on July 1st of each year.
Immigration, Refugees and Citizenship Canada (IRCC) database	Includes demographic information on all immigration application records for people who initially applied to land in Ontario since 1985.
ICES Mother-Baby Linked Database (MOMBABY)	Links the CIHI-DAD inpatient hospital admission records of delivering mothers and their newborns.
Ontario Laboratories Information System (OLIS)	Contains information regarding lab test orders and results from hospitals, community labs, and public health labs across Ontario.
The Better Outcomes Registry & Network (BORN)	Provincial body responsible for collecting pregnancy and newborn information and outcomes data for nearly all births in Ontario, Canada.

eTable 4. Characteristics of the Mothers and Their Singleton Neonates At Birth, Contrasting Neonates With Known vs. Unknown ABO Blood Group Status

All data are shown as a number (%) unless otherwise noted.

Characteristic	Neonates with known ABO blood group (N = 138,207)	Neonates with unknown ABO blood group (N = 586,939)	Standardized difference
Maternal characteristics			
Mean (SD) age, years	31.6 (5.1)	30.8 (5.2)	0.16
Age by group, years			
≤19	1723 (1.2)	10,898 (1.9)	0.05
20 to 24	10,391 (7.5)	59,500 (10.1)	0.09
25 to 29	32,795 (23.7)	157,857 (26.9)	0.07
30 to 34	53,785 (38.9)	219,446 (37.4)	0.03
35 to 39	31,878 (23.1)	114,718 (19.5)	0.09
≥ 40	7635 (5.5)	24,520 (4.2)	0.06
Median (IQR) gravidity	1 (0-2)	1 (0-2)	0.03
Gravidity			
0	46,490 (33.6)	188,839 (32.2)	0.03
1	43,025 (31.1)	185,840 (31.7)	0.01
2	24,382 (17.6)	107,005 (18.2)	0.02
≥ 3	24,264 (17.6)	105,078 (17.9)	0.01
Unknown/Missing	46 (0.1)	177 (0.1)	0.00
Median (IQR) parity	1 (0-1)	1 (0-1)	0.07
Parity			
0	64,072 (46.4)	252,804 (43.1)	0.07
1	48,726 (35.3)	213,937 (36.4)	0.03
2	16,979 (12.3)	79,476 (13.5)	0.04
≥ 3	8430 (6.1)	40,722 (6.9)	0.03
World region of origin			
Canada[a]	94,008 (68.0)	414,691 (70.7)	0.06
Caribbean/Africa	4826 (3.5)	21,861 (3.7)	0.01
East Asia/Pacific	12,577 (9.1)	39,329 (6.7)	0.09
Hispanic America	2962 (2.1)	12,142 (2.1)	0.01
Middle East/North Africa	6175 (4.5)	20,830 (3.5)	0.05
South Asia	10,794 (7.8)	53,984 (9.2)	0.05
Western Nations/Europe	6865 (5.0)	24,102 (4.1)	0.04
Residential income quintile (Q)			

© 2024 Butler EA et al. *JAMA Network Open*

Characteristic	Neonates with known ABO blood group (N = 138,207)	Neonates with unknown ABO blood group (N = 586,939)	Standardized difference
Q1 (lowest), or unknown	27,966 (20.2)	131,587 (22.4)	0.05
Q2	26,360 (19.1)	118,648 (20.2)	0.03
Q3	28,969 (21.0)	122,793 (20.9)	0.01
Q4	31,226 (22.6)	117,846 (20.1)	0.06
Q5 (highest)	23,686 (17.1)	96,065 (16.4)	0.02
Rural residence	9491 (6.9)	60,301 (10.3)	0.12
ABO Blood Group			
A	29,286 (21.2)	--	--
B	14,531 (10.5)	--	--
AB	4423 (3.2)	--	--
O	89,967 (65.1)	--	--
Rh factor negative	32,660 (23.6)	--	--
Maternal conditions within 365 days prior to conception			
Type 1 or type 2 diabetes mellitus	172 (0.1)	587 (0.1)	0.01
Chronic hypertension	94 (0.1)	243 (0.1)	0.01
Sickle cell disease	243 (0.2)	322 (0.1)	0.04
Pre-pregnancy body mass index group, kg/m^2 [b]			
Underweight (< 18.5)	5720 (4.1)	26,221 (4.5)	0.02
Normal (18.5 to 24.9)	57,180 (41.4)	258,844 (44.1)	0.06
Overweight (25.0 to 29.9)	27,092 (19.6)	125,016 (21.3)	0.04
Obese (≥ 30)	21,785 (15.8)	98,978 (16.9)	0.03
Unknown/Missing	26,430 (19.1)	77,880 (13.3)	0.16
Tobacco or substance use	570 (0.4)	1,568 (0.3)	0.03
Any autoimmune disorder	940 (0.7)	2966 (0.5)	0.02
Maternal conditions during the index pregnancy			
Gestational diabetes mellitus among women without type 1 or type 2 diabetes mellitus	12,277 (8.9)	44,977 (7.7)	0.04
Preeclampsia	1519 (1.1)	3,184 (0.5)	0.06
Rh incompatibility	90 (0.1)	99 (0.1)	0.02
Placenta previa	1251 (0.9)	3,983 (0.7)	0.03
Premature rupture of membranes	20,775 (15.0)	71,825 (12.2)	0.08
Group B streptococcus positive[c]	24,646 (17.8)	114,951 (19.6)	0.05

© 2024 Butler EA et al. *JAMA Network Open*

Characteristic	Neonates with known ABO blood group (N = 138,207)	Neonates with unknown ABO blood group (N = 586,939)	Standardized difference
Group B streptococcus antibiotics	24,958 (18.1)	117,633 (20.0)	0.05
Postpartum haemorrhage	7944 (5.7)	27,889 (4.8)	0.05
Mode of delivery			
Vaginal	97,435 (70.5)	419,140 (71.4)	0.02
Caesarian	40,772 (29.5)	167,799 (28.6)	0.02
Newborn characteristics at index birth			
Female	66,691 (48.3)	285,845 (48.7)	0.01
Mean (SD) gestational age, weeks	38.4 (2.4)	38.9 (1.5)	0.26
Median (IQR) Apgar score at 5 minutes[d]	9 (9-9)	9 (9-9)	0.14
Apgar score at 5 minutes, by group[d]			
0 to 3	683 (0.5)	1134 (0.2)	0.05
4 to 7	3013 (2.2)	7947 (1.4)	0.06
≥ 7	130,568 (94.5)	571,447 (97.4)	0.15
Unknown/Missing	3943 (2.9)	6411 (1.1)	0.13
Hereditary immunodeficiency	23 (0.1)	22 (0.1)	0.01
Neonatal jaundice or hyperbilirubinemia	14,290 (10.3)	31,095 (5.3)	0.19
Hemolytic disease of the newborn	2297 (1.7)	3,400 (0.6)	0.10
Admitted to NICU	23,390 (16.9)	65,981 (11.2)	0.16
ABO blood group			
A	41,820 (30.3)	--	--
B	21,556 (15.6)	--	--
AB	4261 (3.1)	--	--
O	70,570 (51.1)	--	--
Rh factor positive	118,403 (85.7)	--	--

[a]Includes long-term residents, residing in Canada more than 10 years.

[b]Comprises 620,869 births (85.6%) with known maternal pre-pregnancy body mass index.

[c]Comprises 628,532 births (86.7%) with known Group B streptococcus status.

[d]Comprises 702,046 births (96.8%) with known Apgar score at 5 minutes.

© 2024 Butler EA et al. *JAMA Network Open*

eTable 5. Characteristics of the Mothers and Their Singleton Neonates At Birth, Contrasting Neonates With a Microbiology Sample Taken vs. Those Who Did Not

All data are shown as a number (%) unless otherwise noted.

Characteristic	Neonates with a microbiology sample taken (N = 11,716)	Neonates without a microbiology sample taken (N = 126,491)	Standardized difference
Maternal characteristics			
Mean (SD) age, years	31.3 (5.4)	31.6 (5.1)	0.07
Age by group, years			
≤19	226 (1.9)	1497 (1.2)	0.06
20 to 24	1077 (9.2)	9314 (7.4)	0.07
25 to 29	2883 (24.6)	29,912 (23.6)	0.02
30 to 34	4304 (36.7)	49,481 (39.1)	0.05
35 to 39	2524 (21.5)	29,354 (23.2)	0.04
≥ 40	702 (6.0)	6933 (5.5)	0.02
Median (IQR) gravidity	1 (0-2)	1 (0-2)	0.05
Gravidity			
0	4515 (38.5)	41,975 (33.2)	0.11
1	3110 (26.5)	39,915 (31.6)	0.11
2	1852 (15.8)	22,530 (17.8)	0.05
≥ 3	2229 (19.0)	22,035 (17.4)	0.04
Unknown/Missing	10 (0.1)	36 (0.1)	0.02
Median (IQR) parity	0 (0-1)	1 (0-1)	0.14
Parity			
0	6360 (54.3)	57,712 (45.6)	0.17
1	3290 (28.1)	45,436 (35.9)	0.17
2	1298 (11.1)	15,681 (12.4)	0.04
≥ 3	768 (6.6)	7662 (6.1)	0.02
World region of origin			
Canada[a]	7767 (66.3)	86,241 (68.2)	0.04
Caribbean/Africa	492 (4.2)	4334 (3.4)	0.04
East Asia/Pacific	1093 (9.3)	11,484 (9.1)	0.01
Hispanic America	230 (2.0)	2732 (2.2)	0.01
Middle East/North Africa	471 (4.0)	5704 (4.5)	0.02
South Asia	1149 (9.8)	9645 (7.6)	0.08
Western Nations/Europe	514 (4.4)	6351 (5.0)	0.03
Residential income quintile (Q)			

© 2024 Butler EA et al. *JAMA Network Open*

Characteristic	Neonates with a microbiology sample taken (N = 11,716)	Neonates without a microbiology sample taken (N = 126,491)	Standardized difference
Q1 (lowest), or unknown	2689 (22.9)	25,277 (20.0)	0.07
Q2	2292 (19.6)	24,068 (19.0)	0.01
Q3	2407 (20.5)	26,562 (21.0)	0.01
Q4	2568 (21.9)	28,658 (22.7)	0.02
Q5 (highest)	1760 (15.0)	21,926 (17.3)	0.06
Rural residence	847 (7.2)	8644 (6.8)	0.02
ABO Blood Group			
A	3393 (29.0)	25,893 (20.5)	0.20
B	1745 (14.9)	12,786 (10.1)	0.15
AB	515 (4.4)	3908 (3.1)	0.07
O	6063 (51.7)	83,904 (66.3)	0.30
Rh factor negative	2098 (17.9)	30,562 (24.2)	0.15
Maternal conditions within 365 days prior to conception			
Type 1 or type 2 diabetes mellitus	31 (0.3)	141 (0.1)	0.04
Chronic hypertension	17 (0.1)	77 (0.1)	0.03
Sickle cell disease	17 (0.1)	226 (0.2)	0.01
Pre-pregnancy body mass index group, kg/m^2 [b]			
Underweight (< 18.5)	476 (4.1)	5244 (4.1)	0.00
Normal (18.5 to 24.9)	4377 (37.4)	52,803 (41.7)	0.09
Overweight (25.0 to 29.9)	2241 (19.1)	24,851 (19.6)	0.01
Obese (≥ 30)	2107 (18.0)	19,678 (15.6)	0.07
Unknown/Missing	2515 (21.5)	23,915 (18.9)	0.06
Tobacco or substance use	61 (0.5)	509 (0.4)	0.02
Any autoimmune disorder	94 (0.8)	846 (0.7)	0.02
Maternal conditions during the index pregnancy			
Gestational diabetes mellitus among women without type 1 or type 2 diabetes mellitus	1115 (9.5)	11,162 (8.8)	0.02
Preeclampsia	261 (2.2)	1258 (1.0)	0.10
Rh incompatibility	13 (0.1)	77 (0.1)	0.02
Placenta previa	248 (2.1)	1003 (0.8)	0.11
Premature rupture of membranes	3350 (28.6)	17,425 (13.8)	0.37
Group B streptococcus positive[c]	1732 (14.8)	22,914 (18.1)	0.09

© 2024 Butler EA et al. *JAMA Network Open*

Characteristic	Neonates with a microbiology sample taken (N = 11,716)	Neonates without a microbiology sample taken (N = 126,491)	Standardized difference
Group B streptococcus antibiotics	2921 (24.9)	22,037 (17.4)	0.19
Postpartum haemorrhage	779 (6.6)	7165 (5.7)	0.04
Mode of delivery			
Vaginal	7075 (60.4)	90,360 (71.4)	0.24
Caesarian	4641 (39.6)	36,131 (28.6)	0.24
Newborn characteristics at index birth			
Female	4,910 (41.9)	61,781 (48.8)	0.14
Mean (SD) gestational age, weeks	35.4 (4.7)	38.7 (1.9)	0.93
Median (IQR) Apgar score at 5 minutes[d]	9 (8-9)	9 (9-9)	0.79
Apgar score at 5 minutes, by group[d]			
0 to 3	388 (3.3)	295 (0.2)	0.24
4 to 7	1408 (12.0)	1605 (1.3)	0.44
≥ 7	9628 (82.2)	120,940 (95.6)	0.44
Unknown/Missing	292 (2.5)	3651 (2.9)	0.02
Hereditary immunodeficiency	15 (0.1)	8 (0.1)	0.05
Neonatal jaundice or hyperbilirubinemia	4681 (40.0)	9609 (7.6)	0.82
Hemolytic disease of the newborn	306 (2.6)	1991 (1.6)	0.07
Admitted to NICU	8834 (75.4)	14,556 (11.5)	1.69
ABO blood group			
A	3850 (32.9)	37,970 (30.0)	0.06
B	2095 (17.9)	19,461 (15.4)	0.07
AB	484 (4.1)	3777 (3.0)	0.06
O	5287 (45.1)	65,283 (51.6)	0.13
Rh factor positive	10,243 (87.4)	108,160 (85.5)	0.06

[a]Includes long-term residents, residing in Canada more than 10 years.
[b]Comprises 111,777 births (80.9%) with known maternal pre-pregnancy body mass index.
[c]Comprises 111,275 births (80.9%) with known Group B streptococcus status.
[d]Comprises 134,264 births (97.2%) with known Apgar score at 5 minutes.

© 2024 Butler EA et al. *JAMA Network Open*

eTable 6. The Top-10 Gram-Positive and Gram-Negative Bacterial Species Among Neonates With a Positive Bacterial Culture From a Blood, Cerebral Spinal Fluid, Lung, or Urine Sample Obtained Within 30 Days of Birth

Gram-positive bacteria	No. of events	Proportion of all Gram-positive bacterial infections (%)	Rank	Gram-negative bacteria	No. of events	Proportion of all Gram-negative bacterial infections (%)
Staphylococcus species other than S. aureus	268	39.9	1	Escherichia coli	296	58.7
Enterococcus species	95	13.6	2	Klebsiella species	74	14.7
Staphylococcus aureus	89	13.4	3	Enterobacter species	53	10.5
Group B Streptococcus	61	9.2	4	Citrobacter species	15	3.0
Bacillus species	57	8.6	5	Serratia marcescens	14	2.8
Streptococcus species other than Group B Streptococcus	44	3.6	6	Haemophilus species	12	2.4
Paenibacillus species	17	2.6	7	Acinetobacter species	9	1.8
Micrococcus species	12	1.8	8	Proteus mirabilis	8	1.6
Lactobacillus species	7	1.1	9	Pseudomonas aeruginosa	6	1.2
Corynebacterium species	< 6[a]	--	10	Stenotrophomonas maltophilia	< 6[a]	--

[e]Fewer than 6 events are suppressed.

© 2024 Butler EA et al. *JAMA Network Open*

eTable 7 (Additional Analysis 2). Odds of an Invasive Gram-Positive or Gram-Negative Newborn Bacterial Infection Within 30 Days After Birth in Relation to Maternal-Newborn Incongruent vs. Congruent ABO Blood Groups

Outcome	Exposure group	No. (%) with outcome	Unadjusted odds ratio (95% CI)[a]	Adjusted odds ratio (95% CI)[a,b]
**No cultured organism**	*Congruent (N = 100,254)*	99,225 (99.0)	1.00 (ref)	1.00 (ref)
	Incongruent (N = 37,953)	37,625 (99.1)	1.00 (ref)	1.00 (ref)
**Gram-positive cultured organism**	*Congruent (N = 100,254)*	512 (5.1)	1.00 (ref)	1.00 (ref)
	Incongruent (N = 37,953)	151 (4.0)	0.78 (0.65 to 0.93)	0.85 (0.71 to 1.02)
**Gram-negative cultured organism**	*Congruent (N = 100,254)*	377 (3.8)	1.00 (ref)	1.00 (ref)
	Incongruent (N = 37,953)	127 (3.4)	0.89 (0.73 to 1.09)	0.96 (0.78 to 1.17)
**Mixed cultured organism**	*Congruent (N = 100,254)*	139 (1.4)	1.00 (ref)	1.00 (ref)
	Incongruent (N = 37,953)	50 (1.3)	0.95 (0.69 to 1.31)	1.00 (0.72 to 1.39)

[a]Calculated using multinomial logistic regression.

[b]Adjusted for infant sex and preterm birth < 37 weeks' gestation.

© 2024 Butler EA et al. *JAMA Network Open*

eTable 8. Number of Invasive Newborn Bacterial Infections Diagnosed Within 30 Days After the Birth by Specimen Location

General specimen source	Specimen source	No. of events	Proportion of all infections (%)
Cerebral Spinal Fluid (CSF)	CSF	55	4.1
Blood	Blood	480	35.4
	Cord blood	14	1.0
Urine	Urine	582	42.9
Lung	Endotracheal tube	221	16.3
	Sputum	< 6[a]	--
	Pleural fluid	< 6[a]	--

[a]Fewer than 6 events are suppressed.

© 2024 Butler EA et al. *JAMA Network Open*

eTable 9 (Additional Analysis 4). Risk of Invasive Newborn Bacterial Infection Within 30 Days of the Index Birth in Relation to Maternal-Newborn Incongruent vs. Congruent ABO Blood Groups, by Culture Source

This analysis groups cerebral spinal fluid, blood and lung samples together, and urine samples separately.

Microbiology culture source	Risk factor assessed	No. (rate per 1000) with outcome	Unadjusted relative risk (95% CI)	Adjusted relative risk (95% CI)[a]
Cerebral spinal fluid, blood or lung	Congruent ABO blood groups (N = 100,254)	595 (5.9)	1.0 (ref)	1.0 (ref)
	Incongruent ABO blood groups (N = 37,953)	180 (4.7)	0.80 (0.68 to 0.94)	0.88 (0.75 to 1.04)
Urine	Congruent ABO blood groups (N = 100,254)	434 (4.3)	1.0 (ref)	1.0 (ref)
	Incongruent ABO blood groups (N = 37,953)	148 (3.9)	0.90 (0.75 to 1.09)	0.95 (0.79 to 1.14)

[a]Adjusted for infant sex and gestational age at birth (in weeks).

© 2024 Butler EA et al. *JAMA Network Open*

eTable 10 (Additional Analysis 5). Risk of the Composite Outcome of Newborn Bacterial Infection or Death Within 30 Days of Birth, in Relation to Maternal-Newborn Incongruent vs. Congruent ABO Blood Groups

Exposure	No. (rate per 1000) with composite outcome	Adjusted relative risk (95% CI)[a]
Congruent ABO blood groups (N =100,254)	1141 (11.4)	1.00 (ref)
Incongruent ABO blood groups (N = 37,953)	373 (9.8)	0.94 (0.83 to 1.05)

[a]Adjusted for infant sex and preterm birth < 37 weeks' gestation.

© 2024 Butler EA et al. *JAMA Network Open*

eFigure 1. Flow Diagram Showing Cohort Creation

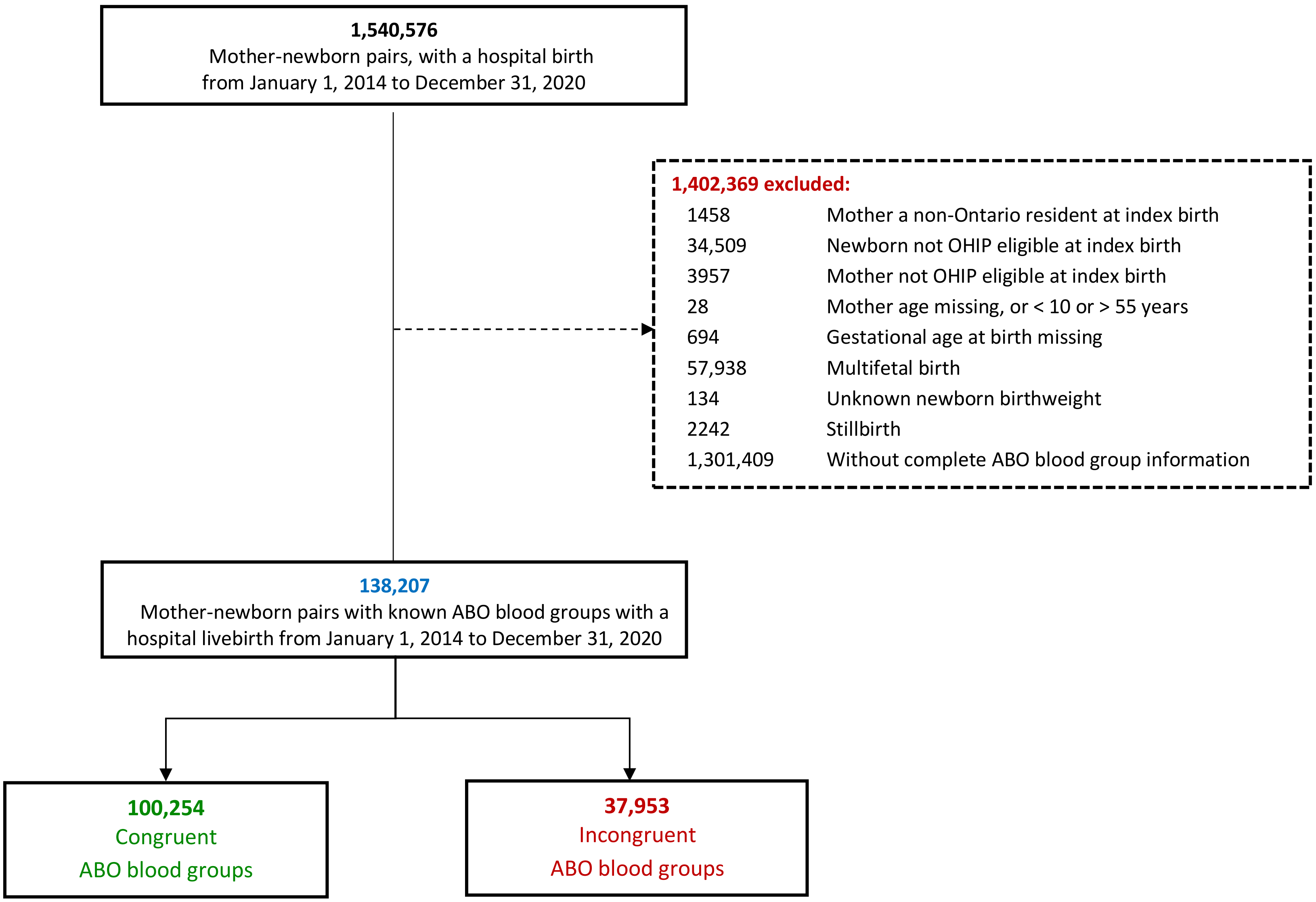

© 2024 Butler EA et al. *JAMA Network Open*

eFigure 2. Conceptual Model of Effect Measure Modifiers and Other Variables

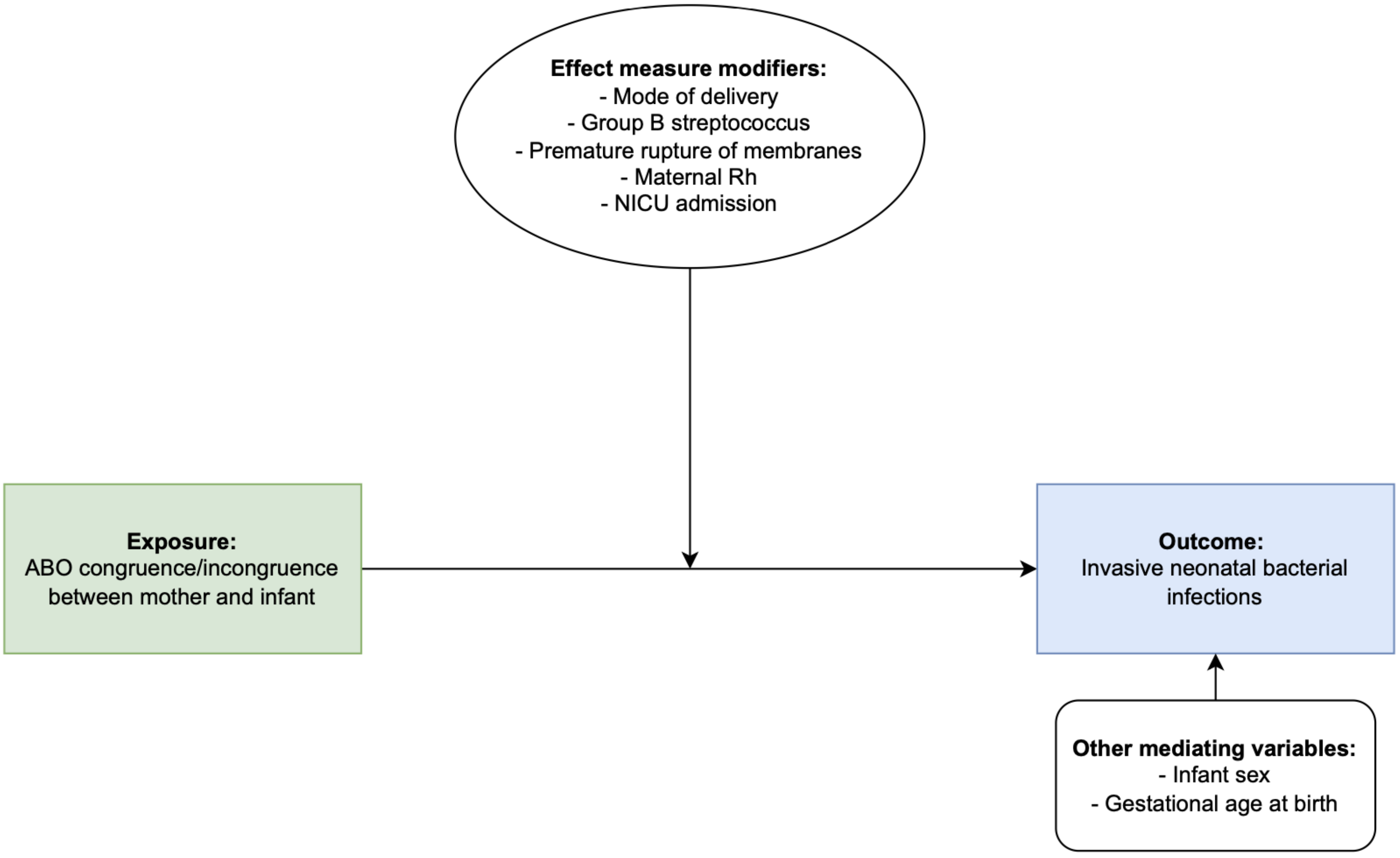

© 2024 Butler EA et al. *JAMA Network Open*
