# Supplementary material for: Maternal-Newborn ABO Blood Groups and Risk of Bacterial Infection in Newborns
Source: JAMA Netw Open. 2024 Oct 30;7(10):e2442227. doi: 10.1001/jamanetworkopen.2024.42227 (PMC11525604; doi:10.1001/jamanetworkopen.2024.42227)
Supplement: Supplement 2. — Data Sharing Statement [file jamanetwopen-e2442227-s002.pdf]

Data Sharing Statement

Butler. Maternal-Newborn ABO Blood Groups and Risk of Bacterial Infection in Newborns. *JAMA Netw Open*. Published October 30, 2024. doi:10.1001/jamanetworkopen.2024.42227

Data
Data available: No
